# Alkylaminophenol Induces G1/S Phase Cell Cycle Arrest in Glioblastoma Cells Through p53 and Cyclin-Dependent Kinase Signaling Pathway

**DOI:** 10.3389/fphar.2019.00330

**Published:** 2019-04-02

**Authors:** Phuong Doan, Aliyu Musa, Nuno R. Candeias, Frank Emmert-Streib, Olli Yli-Harja, Meenakshisundaram Kandhavelu

**Affiliations:** ^1^Molecular Signaling Lab, Faculty of Medicine and Health Technology, Tampere University and BioMediTech, Tampere, Finland; ^2^Institute of Biosciences and Medical Technology, Tampere, Finland; ^3^Predictive Medicine and Data Analytics Lab, Faculty of Medicine and Health Technology, Tampere University and BioMediTech, Tampere, Finland; ^4^Faculty of Engineering and Natural Sciences, Tampere University, Tampere, Finland; ^5^Computaional Systems Biology Group, Faculty of Medicine and Health Technology, Tampere University and BioMediTech, Tampere, Finland; ^6^Institute for Systems Biology, Seattle, WA, United States

**Keywords:** phenol, anticancer, cytotoxicity, apoptosis induction, gene expression, cell cycle

## Abstract

Glioblastoma (GBM) is the most common type of malignant brain tumor in adults. We show here that small molecule 2-[(3,4-dihydroquinolin-1(2H)-yl)(p-tolyl)methyl]phenol (THTMP), a potential anticancer agent, increases the human glioblastoma cell death. Its mechanism of action and the interaction of selective signaling pathways remain elusive. Three structurally related phenolic compounds were tested in multiple glioma cell lines in which the potential activity of the compound, THTMP, was further validated and characterized. Upon prolonged exposer to THTMP, all glioma cell lines undergo p53 and cyclin-dependent kinase mediated cell death with the IC_50_ concentration of 26.5 and 75.4 μM in LN229 and Snb19, respectively. We found that THTMP strongly inhibited cell growth in a dose and in time dependent manner. THTMP treatment led to G1/S cell cycle arrest and apoptosis induction of glioma cell lines. Furthermore, we identified 3,714 genes with significant changes at the transcriptional level in response to THTMP. Further, a transcriptional analysis (RNA-seq) revealed that THTMP targeted the p53 signaling pathway specific genes causing DNA damage and cell cycle arrest at G1/S phase explained by the decrease of cyclin-dependent kinase 1, cyclin A2, cyclin E1 and E2 in glioma cells. Consistently, THTMP induced the apoptosis by regulating the expression of Bcl-2 family genes and reactive oxygen species while it also changed the expression of several anti-apoptotic genes. These observations suggest that THTMP exerts proliferation activity inhibition and pro-apoptosis effects in glioma through affecting cell cycle arrest and intrinsic apoptosis signaling. Importantly, THTMP has more potential at inhibiting GBM cell proliferation compared to TMZ, the current chemotherapy treatment administered to GBM patients; thus, we propose that THTMP may be an alternative therapeutic option for glioblastoma.

## Introduction

Glioblastoma (GBM) is known as the most aggressive primary brain tumor. Although different treatments have been combined such as surgical operation, chemotherapy, or radiotherapy, no standard treatment has been proven to be effective for treating brain tumor. It is noted that patients with glioblastoma have an average survival of 12–15 months. For chemotherapy, temozolomide (TMZ) is one of the drugs accepted to be used in combination with radiotherapy to treat brain tumor (Stupp et al., 2005). However, some limitations related to use of TMZ such as the over expression of O6-methylguanine-DNA methyltransferase (MGMT) and/or lacking of a DNA repair pathway in GBM cells (Hegi et al., 2005) still need to be addressed; therefore, effective recurrence needs to be explored further.

A comprehensive understanding of the response of glioblastomas to chemotherapy and detailed chemotherapy resistance analysis of gliomas may help to identify effective agents for the treatment of this disease. Currently, many chemical compounds including sorafenib (Yang et al., 2010), bevacizumab (Friedman et al., 2009), and kaempferol (Sharma et al., 2007) have been studied for anti-glioma ability, especially for inducing GBM growth arrest and apoptosis. In the past few decades, many efforts have been made in understanding chemotherapy-induced DNA damage response (DDR) such as activation of checkpoint, repair and cell death pathways. It is reported that GBM responds to DNA damage induced by genotoxic drugs by activating DNA repair machinery (Erasimus et al., 2016). Thereby, improving chemotherapy response should be made to address this issue. Beside the DNA damage, targeting cell cycle arrest and apoptosis also grasped the attention for GBM treatment. In glioma cells, several key regulatory elements of cell cycle and apoptosis alter the expression of cyclin-dependent kinases such as Bcl-2 protein family, p53 protein, inhibitor of apoptosis proteins (IAPs) or receptor tyrosine kinases like the epidermal growth factor receptor (EGFR) and their down-stream signaling cascade. Among these signaling pathways, p53 plays an essential role in cellular responses to DNA damage and regulation of cell cycle and apoptosis. It is well known that p53 functions as a transcription factor for genes relevant for the regulation of the cell cycle (e.g., p21) or apoptosis (e.g., BAX, BAK, PUMA, Bcl-2). Furthermore, p53 may also promote apoptosis through transcription-independent mechanisms and direct interactions with members of the Bcl-2 family of proteins in the cytosol or mitochondria.

In the past decades, many advances have been made in understanding the ability of phenolic compounds in acting as effective chemopreventive agents especially throughout the properties of inducing cell cycle arrest and apoptosis in tumor cells (Wu et al., 2009). Several mechanisms were studied explaining the effectiveness of these compounds as chemopreventive agents for cancer treatment. These compounds can suppress the overexpression of pro-oxidant enzymes implicated in the development of cancer. They are also able to inhibit the transcriptional factor activation, thus regulating target genes correlated with cell survival, apoptosis and proliferation (Wcislo et al., 2013). For instance, polyphenols have the ability to modulate various targets of apoptosis pathways including the expression of regulatory proteins, cytochrome *c*, activation of caspase 9 and caspase 3 (Selvendiran et al., 2006), increase of caspases-8 and t-Bid levels (Selvendiran et al., 2006), increase of Bax and Bak expression (Selvendiran et al., 2006), down-regulation of Bcl-2 and Bcl-XL expression, and modulation of transcription factor NF-κB (Gong et al., 2003). In addition, a study of resveratrol, a natural phenol, revealed the ability to prevent or delay the onset of several types of cancers because they can regulate multiple cellular processes associated with carcinogenesis. In detail, this compound can inhibit cell proliferation and induce apoptosis by dysregulating cell cycle (Gali-Muhtasib et al., 2015), increasing caspase activity (Kim et al., 2003), and decreasing Bcl-2 and Bcl-XL levels.

Alkylaminophenols, being Mannich bases, are a particular kind of phenols (Roman, 2015). Although reported as precursors of quinone methides (Weinert et al., 2006), which can react with biomacromolecules (Thompson et al., 1993), alkylaminophenol moiety is also found in some FDA-approved drugs namely, amodiaquine, used for malaria treatment (Olliaro et al., 1996) and in topotecan, a topoisomerase inhibitor chemotherapeutic agent (Pommier, 2006). Previously, we reported the potential anticancer activity as apoptosis inducer of several alkylaminophenols on osteosarcoma cells, namely: *N*-[2-hydroxy-5-nitrophenyl(4′-methylphenyl)methyl]indoline (HNPMI) (Doan et al., 2016), 2-[(1,2,3,4-tetrahydroquinolin-1-yl)(4-methoxyphenyl)methyl]phenol (THMPP) (Karjalainen et al., 2017) and 2-[(3,4-dihydroquinolin-1(2H)-yl)(p-tolyl)methyl]phenol (THTMP) (Neto et al., 2016). To our knowledge, the anticancer activity of various phenolic derivatives have been evaluated on several human cancer cell lines but the effect as well as the in depth mechanism of phenols on brain cancer are not well investigated. Motivated by the numerous reports on the anticancer properties of phenolic compounds and our previous studies on alkylaminophenols, we recently examine the effect of HNPMI, THMPP, and THTMP on multiple glioblastoma cell lines (1321N1, LN229, and Snb19). Several *in vitro* preclinical assays were performed to indicate the cytotoxicity of this derivative on GBM. Specifically, the ability to kill GBM cells.

In spite of the multiple mechanisms have been proposed for chemotherapeutic resistance in glioblastoma cells, the analysis of molecular signaling events is still not comprehensive. To date, advances in high-throughput sequencing methodology have provided a large amount of information regarding gene expression at the transcriptome level, as well as the underlying molecular events in response to chemotherapeutic drugs. Hence, the RNA-seq technique was used in this work to investigate alkylaminophenol -responsive genes in GBM cells. Here, we compared the gene expression profile of GMB cells between an alkylaminophenol and temozolomide. After determining the gene expression profile, we focused on the cell cycle arrest and the apoptosis pathway activated by our alkylaminophenol and investigated the significant of cell cycle genes as well as pro-apoptosis and anti-apoptosis genes in gliomas chemotherapeutic resistance. The cell cycle arrest was then validated by FUCCI biosensor and the apoptosis induction validation was performed using Annexin V and PI double staining. Moreover, ROS production and caspase 3/7 activation measurements were conducted to reconfirm the involvement of apoptosis pathway when the GBM cells were treated with phenolic derivatives.

## Materials and Methods

### GBM Cell Lines and Chemical Preparation

1321N1 is a human astrocytoma cell line isolated as a sub clone of the cell line 1181N1 which in turn was isolated from the parent line U-118 MG (one of a number of cell lines derived from malignant gliomas). LN229 cell line was taken from a patient with right frontal parieto-occipital glioblastoma. The cells exhibit mutated p53 (TP53) and possible homozygous deletions in the p16 and p14ARF tumor suppressor genes. Snb19 is a malignant glioblastoma cell line initiated from the surgical resection of a left parietooccipital glioblastoma multiforme tumor. This line has been shown by DNA profiling studies to be a derivative of the U-373 cell line.

Synthesis and spectral characterization of compounds HNPMI (18), THMPP (19), and THTMP (20) were previously reported. These compounds and TMZ (Sigma-Aldrich, United States) were dissolved in dimethyl sulphoxide (DMSO, Sigma-Aldrich, St. Louis, MO, United States) to obtain a stock of 100 mM, from which, intermediate dilutions were prepared. The final concentrations used were 100, 75, 50, 25, and 10 μM, in the culture medium.

### Cell Culture

The human glioma cell lines Snb19, LN229, and mouse embryonal fibroblast (MEF) cell lines were cultured in Dulbecco’s Modified Eagle Medium (DMEM) supplemented with 10% FBS, 0.1 mg/ml Streptomycin, 100 U/ml Penicillin, and 0.025 mg/ml Amphotericin B. For 1321N1 cell line, the culture medium was prepared as previously but it was supplemented with 2 mM sodium pyruvate. HEK293T cells were cultured in DMEM supplemented with 10% FBS, 0.1 mg/ml Streptomycin, 100 U/ml Penicillin, 2 mM sodium pyruvate, and 0.025 mg/ml Amphotericin B. The culture was maintained at 37°C in a humidified atmosphere containing 5% CO2. All of the components for cell culture were purchased from Sigma-Aldrich, St. Louis, MO, United States.

### *In vitro* Cytotoxicity Assay

Cytotoxicity assay was performed to evaluate cell growth inhibition of the three compounds HNPMI, THMPP, and THTMP at 100 μM concentration on three glioblastoma cell lines (1321N1, Snb19, and LN229). Cells were seeded with an initial density of 1 × 10^5^ cells/well in 12-well plates containing appropriate medium for each cell line. When the cells reach 60–70% of confluence, the cells were then treated with the three compounds at 100 μM and incubated for 24 h at culture conditions. Treated cells were collected using centrifugation at 3000 rpm for 10 min. Number of live and dead cells were determined using trypan blue solution and Countess II FL Automated Cell Counter (Thermo Fisher Scientific). Inhibition percentage was calculated using the formula (1). In this experiment, biological and technical replicates were conducted for each condition. Temozolomide (TMZ, Sigma-Aldrich, St. Louis, MO, United States) and DMSO 2% were used as positive and negative control, respectively.

(1) $$\text{Inhibition }(\%)=\frac{\text{Mean No. of untreated cells }(\text{DMSO control})-\text{Mean No. of treated cells}}{\text{Mean No. of untreated cells }\left(\text{DMSO control}\right)}\times 100$$

The cytotoxicity of the top compound was evaluated on multiple GBM cell lines, 1321N1, LN229, Snb19, and HEK293T (immortal cells) human embryonic kidney and normal brain cells MEF. Ten micromolar concentration of the top compound was used to treat the cells followed by trypan blue exclusion assay to quantify the percentage of live and dead cells. The inhibition percentage was calculated as described above.

### Inhibitory Kinetic Study

The inhibitory kinetic study was performed for 24 h exposure time using different concentrations 100, 75, 50, 25, 10 μM of the top compound on 1321N1, Snb19, and LN229 cells. After treatment, the cells were collected as described in the cytotoxicity assay. The positive control TMZ was also utilized. After that the dose-response curves were plotted. Half maximal inhibitory concentration (IC_50_) was calculated based on the curves fit. The two cell lines with best IC_50_ were selected for further time-dependent study. In this study, the cells were treated with IC_50_ concentration of the top compound and incubated for 48 and 72 h. The time-dependent graph was plotted.

### Illumina Sequencing and Bioinformatics Analysis

To perform the RNA-seq, RNA of samples had to be isolated. LN229 and Snb19 cells were seeded into 6 well-plate and incubated overnight. The cells were treated with THTMP and TMZ for 24 h at IC_50_ concentration. The total RNA of the cells were isolated using GeneJET RNA Purification Kit (Thermo Fisher Scientific) following the manufacture’s instructions. Then, the total RNA of 18 samples of LN229 and Snb19 cells (including triplicates of THTMP treated, TMZ treated and untreated samples) were sent to whole transcriptome sequencing by Biomedicum Functional Genomics Unit (FuGU, University of Helsinki, Finland) using Illumina NextSeq 500. The sequencing produced data in bcl format which was converted into FASTQ file format.

### RNASeq Data Analysis Pipeline

FastQC (Andrews, 2010) (version 0.11.2) was used for quality control to ensure that the quality value was above Q30. The Human (homo sapiens) genome FASTA file^1^ and gene annotation GTF file (Homo sapiens human release 92^2^) were obtained from Ensembl. Although RNA-seq is a popular research tool, there is no gold standard for analyzing RNA-seq data. Among the available tools, we chose up-to-date open source tools for mapping, retrieving read counts, and differential expression analysis. We used STAR (Dobin et al., 2013) (version 2.6) to generate indexes and to map reads to the human genome. For assembly, we chose SAMtools (Li et al., 2009) (version 1.2) and the “union” mode of HTSeq (Anders et al., 2014) (version 0.9.1), as the gene-level read counts could provide more flexibility in the differential expression analysis. Both STAR and HTSeq analyses were conducted using the high-performance research computing resources provided by TUT TCSC Merope computing cluster^3^ in the Linux operating system (version 2.6.32). Differential expression (DE) and statistical analysis were performed using DESeq2 (Love et al., 2014) (release 3.3) in R (version 3.2.4). DESeq2 was chosen as a leading statistical method^4^. DESeq2 internally corrects for library size, so it is important to provide un-normalized raw read counts as input. We used variance stabilizing transformation to account for differences in sequencing depth. *P*-values were adjusted for multiple testing using the Benjamini-Hochberg procedure (Benjamini and Hochberg, 1995). A false discovery rate adjusted *p*-value (i.e., *q*-value) <0.05 was set for the selection of DE genes.

### Gene Ontology (GO) and Pathway Analysis

Gene ontology (Ashburner et al., 2000) and KEGG pathway (Kanehisa and Goto, 2000) analyses were performed with the PANTHER over-representation Test (released on Feb 03, 2018) in PANTHER version 13.1^5^ (Mi et al., 2009; Emmert-Streib and Glazko, 2011). This program supports the human genome. PANTHER uses a binomial test and a Bonferroni correction for multiple testing and displayes *z*-scores to indicate whether a potential regulator is activated or inhibited. We used the default settings for statistical analysis in both the PANTHER pathway and GO terms. In the analyses, only pathways and GO terms with *p*-value <0.05 and fold change of 1.5 were set as cutoff values.

### Analysis of Cell Cycle Progression

The Snb19 and LN229 cells were cultured in 96-well plates with the initial density of 1 × 10^5^ cells/well. Cells were incubated overnight with appropriate culture conditions. When the cell confluence reached 60%, they were treated with the IC_50_ concentration of the top compound for 8 h. Then Premo FUCCI Cell Cycle Sensor ^∗^BacMam 2.0^∗^ (Thermo Fisher Scientific) was added into each well and incubated for 16 h following the manufacture’s protocol. The cells were then captured using confocal microscope. The analysis of images was done based on different fluorescent colors of the cells in which red fluorescent cells were the cells in G1 phase, green fluorescent cells means the cells in S, G2, M phase and the overlaid red and green fluorescent cells are the cells in G1/S phase (Zielke and Edgar, 2015).

### Annexin V-FITC/PI Apoptotic Assay

To determine the apoptosis and/or necrosis of the top compound on Snb19 and LN229 cell lines, the Dead Cell Apoptosis Kit with Annexin V FITC and PI (Thermo Fisher Scientific) was used. The apoptosis determination was performed followed by the standard protocol from the manufacture. Briefly, the cells were cultured in 6 well-plate with the initial density of 5 × 10^5^ cells/ well. The cells were treated with IC_50_ concentration of the top compound, TMZ and negative control (DMSO) were harvested and washed in cold PBS. The cell pellets were then resuspended in 1× annexin-binding buffer provided in the kit. Then, 5 μL of FITC conjugated Annexin V and 1 μL of the 100 μg/mL PI working solutions were added to the 100 μL of cell suspension. The cells were incubated at room temperature for 15 min prior to the fluorescence measurements. The image acquisition was done by using EVOS imaging system (Thermo Fisher Scientific) with 20 × objective magnification.

### Detection of Intracellular Reactive Oxygen Species

The Snb19 and LN229 cells were cultured in 12-well plates with the initial density of 1 × 10^5^ cells/well. Cells were incubated overnight with appropriate culture conditions then treated with the IC_50_ concentration of the top compound and TMZ for 5 h. After that, cells were harvested by centrifugation at 3000 rpm for 10 min and transferred into 96-well plate. Cells were incubated with 2 μM 2′,7′-dichlorodihydrofluorescein diacetate (H2DCFDA), known as dichlorofluorescin diacetate (Sigma-Aldrich, St. Louis, MO, United States), for 30 min at cell culture conditions. The cells were then washed with pre-warm PBS and recovered in pre-warmed completed medium for 20 min prior the fluorescence measurement. Fluorescence intensity was measured using plate reader (Fluoroskan Ascent FL, Thermo Labsystems) at excitation 485 nm and emission 538 nm. DMSO and hydrogen peroxide 200 μM were used as the negative and positive controls. The fold increase in ROS production was calculated using the following formula (2).

(2) $$\text{Fold increase=}\frac{F_{test}-F_{blank}}{F_{control}-F_{blank}}$$

Where: *F*_test_ is the fluorescence readings from the treated wells, *F*_control_ is the fluorescence readings from the untreated wells, and *F*_blank_ is the fluorescence readings from the unstained wells.

### Caspases 3/7 Activities Assay

Snb19 and LN229 cells were seeded on 96 well-plates at the initial density of 1 × 10^4^ cells/well with appropriate medium. After culturing for 24 h, cells were treated with TMZ and the top compound at IC_50_ concentration for 5 h. Determination of caspase activity was performed using Caspase-Glo 3/7 Assay kit (Promega, Madison United States) followed by the standard protocol from the manufacture. Briefly, the plate containing cells were removed from incubator and allowed to equilibrate to room temperature for 30 min. An amount of 100 μl of Caspase-Glo reagent was added to the plate containing 100 μl of treated cell, untreated cell, blank or TMZ. After that, content of wells was gently mixed using a plate shaker at 300–500 rpm for 30 s. The plate was incubated for further 1 h before measuring the luminescence using a plate-reading luminometer (Fluoroskan Ascent FL, Thermo Labsystems). The fold increase in caspase 3/7 was calculated using formula (2) as described in ROS assay.

### Statistical Analysis

All of the experiments were conducted with three biological repeats and technical repeats. The data was analyzed using SPSS 20.0. For comparison between the tested groups, statistical significant differences were evaluated with the *t*-test using a threshold of *P* < 0.001 and *P* < 0.05. For comparison of more than two groups, statistical significance was determined with a one-way ANOVA test with the level of significance at *p* < 0.05.

## Results

### Characterization of Human Glioma Cells Treated With Alkylaminophenols

**FIGURE 1:**
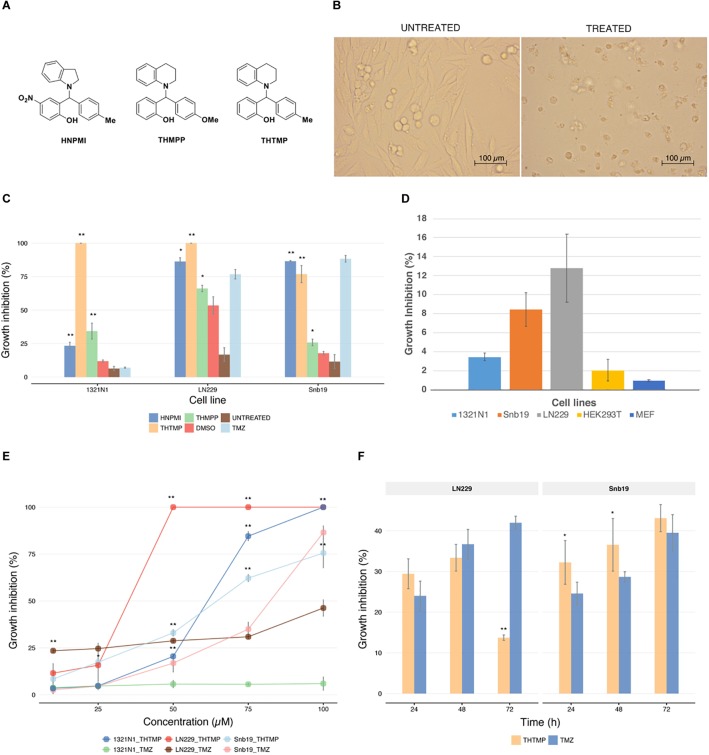
Effect of alkylaminophenols characterized by cell growth arrest. **(A)** Molecular structure of three tested phenolic compounds (HNPMI, THMPP, and THTMP). **(B)** Demonstrated images of morphological changes in GBM cells at 24 h after treatment. **(C)** Cell growth inhibition was determined with trypan blue solution for compounds HNPMI, THMPP, THTMP, and TMZ against GBM cells (1321N1, LN229, and Snb19) at 100 μM concentration. **(D)** Growth inhibitory effect of THTMP on different cell lines 1321N1, LN229, and Snb19 and HEK293T cells at 10 and 100 μM at 24 h post-treatment. **(E)** Effect of THTMP and TMZ on GBM cell growth. Different concentrations including 10, 25, 50, 75, and 100 μM were utilized and incubated for 24 h. **(F)** Time-dependent effect of THTMP and TMZ on LN229 and Snb19 at 24, 48, and 72 h post-treatment at IC50 concentrations. All experiments were performed with three biological repeats and two technical repeats. ^∗∗^*P* < 0.001, ^∗^*P* < 0.05 compared to the TMZ.

Three GBM cell lines were treated with 100 μM HNPMI, THMPP, and THTMP (Figure 1A). After 24 h of treatment, the cells lost the proliferative activity with dramatic changes in morphology, losing attachment property and incrementing granularity (Figure 1B). Delightfully, THTMP strongly inhibited the growth of GBM cells 1321N1, LN229, and Snb19 (Figure 1C). At 100 μM, THTMP was responsible for almost 100% cell death of 1321N1 and LN229 and approximately 80% cell death of Snb19. HNPMI also showed high cytotoxicity on LN229 and Snb19 with more than 80% cell death while it had little effect on 1321N1 with only 23% cell death. THMPP has the least cytotoxicity effect compared to THTMP and HNPMI (Figure 1C).

From the above results, it is concluded that THTMP is a potent inhibitor of GBM cell growth. Here, we also used an immortal cell line, HEK293T and a non-tumorous cell line, MEF to examine the effect of THTMP. In general, THTMP has higher cytotoxicity effect on GBM cells compared with immortal and non-tumorous cells. In which, approximately 3 to 12% cell death were found in different GBM cell lines whilst only 2 and 1% growth inhibition were observed in HEK293T and MEF cells, respectively (Figure 1D). Thus, this result suggests that THTMP has the selectivity on GBM cells and was hence selected for further studies.

The dose-dependent inhibitory effect of THTMP against GBM cells was studied at 10, 25, 50, 75 and 100 μM concentrations (Figure 1E). Among three cell lines, LN229 was the most affected by THTMP with an IC_50_ concentration of 26.5 ± 0.03 μM, followed by 1321N1 with an IC_50_ of 61.9 ± 0.65 μM and least inhibited cell line was Snb19 with an IC_50_ of 75.5 ± 2.18. Besides, TMZ showed better effect on Snb19 than LN229 while seemly no effect was observed in 1321N1. This is in an agreement with the previous findings (Lee, 2016).

Based on the results obtained for THTMP and TMZ in dose response curve, further studies were performed on LN229 and Snb19 to understand the compound action mode as anticancer drug. To observe the effect of THTMP over the hours on cell viability, LN229 and Snb19 cells were treated for 24, 48, and 72 h with IC_50_ concentration (Figure 1F). The result showed that there was a time-dependent effect on Snb19 from 24 to 72 h and on LN229 from 24 to 48 h. In details, the growth inhibition of Snb19 was increased from 32.2 to 36.5% and to 43.1% at 24, 48, and 72 h post-treatment, respectively. The growth inhibition of LN229 was increased from 29.4% at 24 h treatment to 33.4% at 48 h treatment and was decreased to 13.7% at 72 h treatment.

### Global Change in Gene Expression in Response to Top Compound

#### Principal Component Analysis (PCA) and Hierarchical Clustering Analysis

**FIGURE 2:**
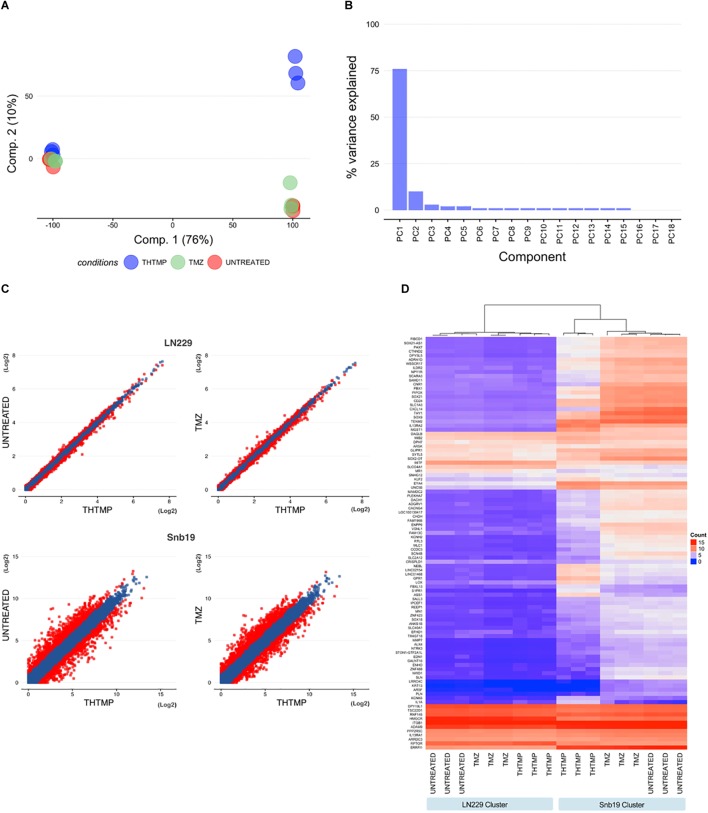
Hierarchical clustering analysis, heatmap, and principal component analysis. **(A)** Principal component analysis (PCA) for all samples measured from LN229 and Snb19 cell lines. Principal component 1 (PC1) and principal component 2 (PC2) were identified by variance stabilizing transformation in DESeq2 on cell line samples. (Red, Untreated samples; Green, Temozolomide samples; Blue, THTMP samples). **(B)** The figure shows the percentage of variance that indicates how much variance was explained by PC1 and PC2. **(C)** Scatter plot analysis of gene expression using comparisons (THTMP vs. Untreated and THTMP vs. TMZ) in LN229 and Snb19 cell lines from the GMB. Genes whose expression levels changed by more than 1.5-fold after treatment are indicated in red. **(D)** Hierarchical clustering analysis and heatmap of the 100 genes with the smallest q-values in the time course analysis in DESeq2 (for Untreated, Temozolomide, and THTMP designate three replicate samples; negative, positive, and compound are individual cell lines in each group).

We performed PCA at each sample to determine whether samples in each cell line group clustered with each other or other groups. First, we used HTSeq to count reads that uniquely aligned to one gene, and these data were then imported into DESeq2 to generate PCA plots (Figure 2A). Furthermore, PCA scree plots confirmed that principal components 1 (PC1) and 2 (PC2) accounted for 70–80% of the total variation in gene expression at each time point (Figure 2B). To further investigate the cell-type dependent nature of the DEGs, we performed hierarchical clustering of the top 100 DEGs (i.e., those with the smallest *q*-values identified in the cell line analysis in DESeq2). In agreement with the PCA plots, this analysis demonstrated clustering of almost all sample groups from each cell line forming two clusters (Figure 2D).

#### Differentially Expressed Genes (DEGs)

**FIGURE 3:**
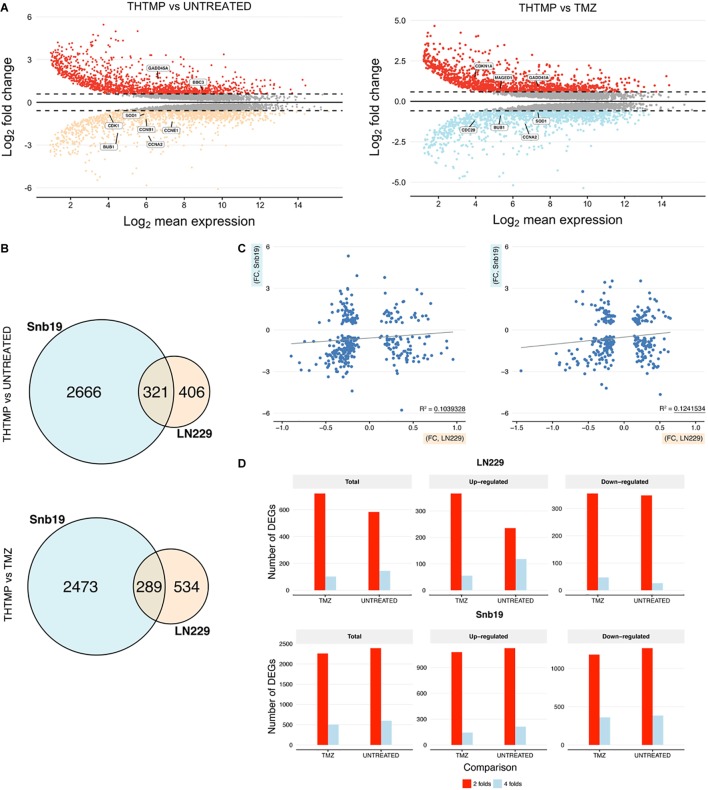
DEGs comparisons on LN229 and Snb19 samples. **(A)** MA-plot from means and log fold changes. The figure shows differential gene expression from the two inter-group comparisons (THTMP vs. Untreated; THTMP vs. Temozolomide). For MA-plot construction, a gene was considered to be differentially expressed between groups at an absolute log2 fold change >1.5 or<-1.5 and a *q*-value of 0.05 (moderated *t*-test; Benjamini-Hochberg procedure). **(B)** Overlapping DEGs in LN229 and Snb19 samples compared with positive and negative control at C1 and C2. For each comparison, only genes with a *q*-value <0.05 were considered as DEGs. The number of DEGs found at each comparison are indicated. **(C)** Scatter plots of fold-changes in gene expression levels after treatment of C1 and C2. The R^2^ value was calculated for genes with *t*-test *p*-values <0.1. **(D)** The total number and upregulated/downregulated number of DEGs of both cell lines after treatment.

In average, 20,090 genes were mapped by at least one read in each of the two cell line samples. Overall, 7,299 DEGs with a *q*-value <0.05 and fold change >1.5 (LN229 1,550; Snb19 5,749) were detected over the two comparisons (C1: THTMP vs. Untreated; C2: THTMP vs. TMZ) in the cell type analysis of DESeq2 (Supplementary Tables S1–S4). The results of plot analysis of gene expression in two cell lines of the GBM after treatment are shown in Figure 2C. The numbers of differentially expressed genes with more than 1.5-fold change were higher in Snb19 than in LN229 (Figure 3D). Indeed, there were higher number of differentially expressed genes in these Snb19 cell line when compared with LN229 cell line as shown in Figure 2C. We applied the MA plot function in DESeq2 to visualize the top genes with the smallest *q*-values (Figure 3A). We investigated the similarity in differential gene expression profiles regulated LN229 and Snb19. The fold-changes in overlapped genes filtered by the *q*-value <0.05 were plotted for LN229 and Snb19 cell lines. Comparison of gene expression profiles showed correlations between LN229 and Snb19 cell lines (*R*^2^ > 0.10, Figure 3C left in C1; *R*^2^ > 0.12, Figure 3C right in C2). Venn diagrams indicated overlap in genes whose expression was regulated in the same direction (Figure 3B). We identified 3,714 DEGs between THTMP and untreated (negative control) samples among the cell lines (*q*-value <0.05) (Supplementary Tables S1–S4 and Figure 3B top). In this comparison, Snb19 demonstrated the most DEGs, with 321 of the 3,714 DEGs common to both LN229 and Snb19. We also compared the THTMP and TMZ samples as a positive control group, both individually and combined as a single “affected” group. In these comparisons, 3,585 number of DEGs were identified, with the largest number of DEGs identified in Snb19 cell line, and 289 out of 3,585 DEGs common in both cell lines (Supplementary Tables S1–S4 and Figure 3B down).

A substantial overlap of DEGs was present when comparing LN229 and Snb19 samples with the control group at C1 and C2. The overlapping DEGs between the cell line were higher at C1 (3,714 DEGs) than at C2 (3,585 DEGs), supporting the two groups behave similarly at the end-stage of the treatment, as expected based on Figure 3B. Both cell lines shared appreciable proportions of gene expression profiles (21.23% of genes in LN229; 78.76% of genes in Snb19). The complete lists of DEGs from the cell line analysis and all pairs of comparisons appear in Supplementary Tables S1–S4.

### Dynamic Cellular Damage Responses Induced by THTMP

**FIGURE 4:**
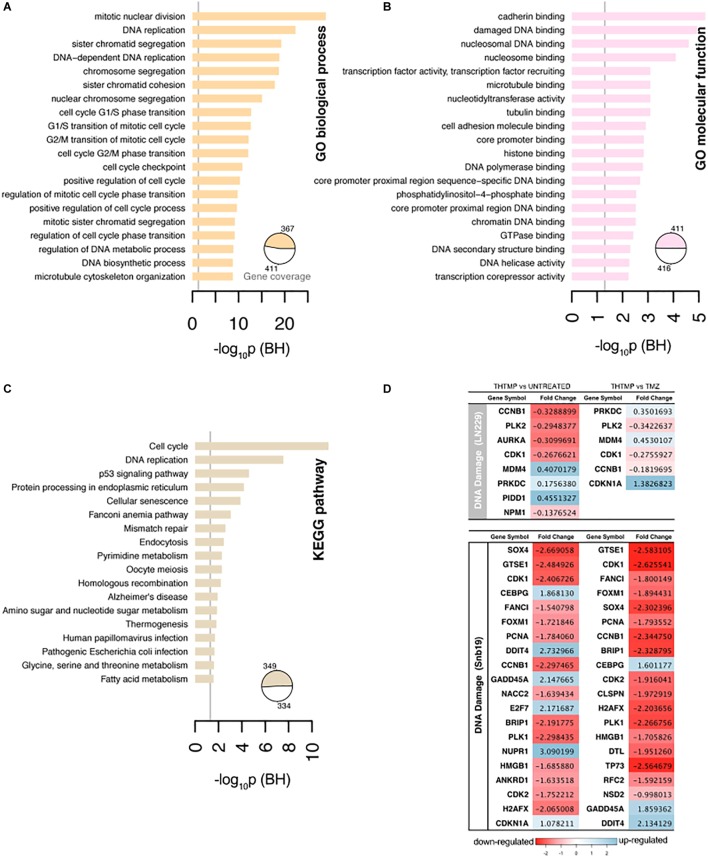
Selected results of Gene Ontology terms and pathways over-representation analysis (FDR < 0.01) on the top 20 terms. **(A)** Overrepresented Gene Ontology (GO) Biological process terms. **(B)** Overrepresented GO Molecular function terms. **(C)** Overrepresented KEGG pathway terms. The x-axis contains the number of genes involved in a particular pathway that were found differentially expressed in our study. The pie charts indicate the fraction of the signature genes associated with significantly enriched terms. The number of genes were normalized to allow comparisons between groups within the same cell line, and the vertical line on the pathways corresponds to the significant *p*-values. **(D)** The top 20 DEGs which are involved in the DNA damage on LN229 and Snb19. The DEGs were color coded, with the colors corresponding to the up- and down- expressed.

The gene ontology was conducted to analyze up and down regulated genes regarded to DNA damage. GO analysis identified the list of genes that were enriched in DNA replication, sister chromatid segregation, DNA-dependent DNA replication, chromosome segregation, sister chromatid cohesion, and nuclear chromosome segregation process. These biological processes are involved in the DNA replication pathway in both cell lines when they were treated with THTMP and TMZ (Figure 4A). Enrichment analysis for GO molecular function and pathways clearly demonstrated related phenotypes associated with GBM (Figure 4B,C). GO terms cadherin binding, damaged DNA binding for molecular function appeared to be significantly overrepresented, and none significantly underrepresented. Cadherin binding, a type I membrane protein involved in cell adhesion and damaged DNA binding, interacting selectively and non-covalently with damaged DNA have coordinated effect on regulation and function in DNA damage (Daido et al., 2005). Previous studies have shown that GBMs are highly resistant to single inhibitor, suggesting that combinational strategies involving standard chemotherapies like TMZ and pathway inhibitors might be a possible future direction for treating GBM (Jacinto and Esteller, 2007).

Genes associated with the DNA damage were listed in the Figure 4D. In general, more DEGs were observed in Snb19 when they were treated with THTMP and TMZ. Here, the top 20 DEGs were listed in Figure 4D. In LN229, eight DEGs were expressed when they were treated with THTMP and six DEGs were found in TMZ treatment. CDK1 gene is downregulated when the cells were treated with THTMP and TMZ in both cell types. It is reported that CDK1 was observed to be enriched in the p53 signaling pathway, which is induced by a number of stress signals, including DNA damage, oxidative stress and activated oncogenes. It is noted that p53 signaling network is an integral tumor suppressor pathway in GBM pathogenesis that affects cellular processes, including cell cycle control and cell death execution (Stegh et al., 2010). Moreover, CDKN1A was found to be upregulated in Snb19 when they were treated with THTMP (Figure 4D). It is noted that CDKN1A is a gene encoding for p21 protein which contributes to the cell response to DNA damage not only by inactivating G1-phase cyclins/CDKs complexes, but also through other processes, which possibly include direct interaction with PCNA to inhibit DNA replication, and indirect effects mediated by interaction with other cell cycle regulators.

Thereby, our result suggests that DNA damage has been confirmed by the downregulation of CDK1 as well as upregulation of CDKN1A leading to activation of p53 and p21 signaling; thus, inhibiting the growth of glioblastoma. Moreover, CDK1 also plays an important role in cell cycle control (Santamaría et al., 2007). Here, the downregulation of CDK1 expression was identified in THTMP treated conditions confirming cyclin-dependent kinase mediated cell cycle arrest. Detailed investigation of cell cycle arrest was performed using biosensor and gene expression profiling.

### THTMP Induces G1/S DNA Damage Checkpoint

**FIGURE 5:**
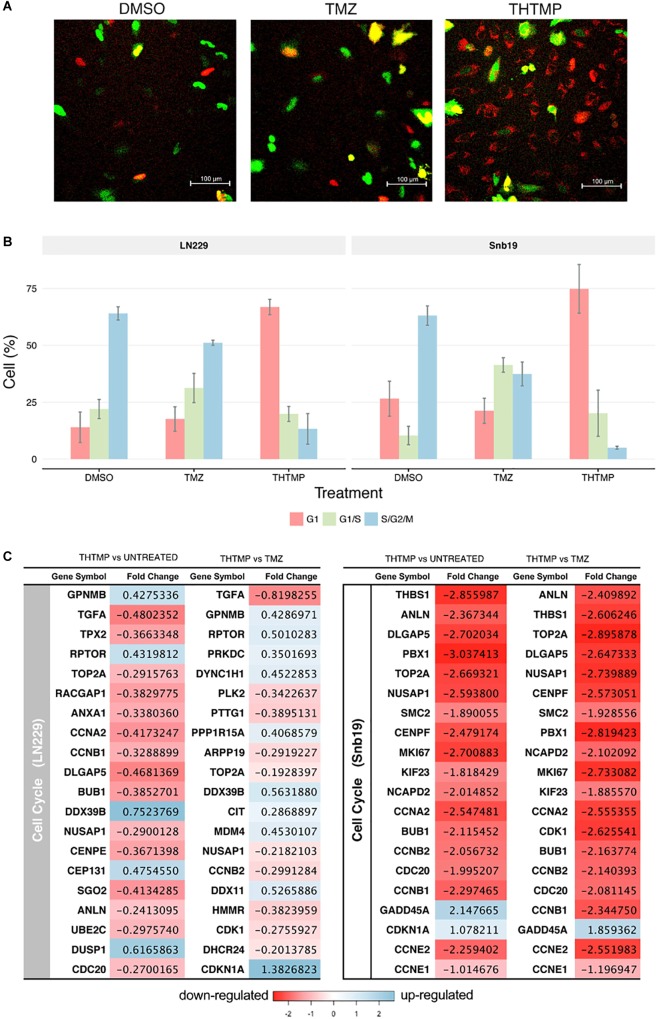
Cell cycle analysis using FUCCI and RNA-seq. **(A)** Demonstrated images of cell which were dyed with FUCCI in different conditions (DMSO, TMZ, and THTMP). Red cells corresponding to the cells in G1, yellow cells corresponding to the cells in G1/S phase and the green cells corresponding to the cells in S/G2/M phase **(B)** Percentage of total cell in different phases when they were treated with THTMP and TMZ. **(C)** The top 20 DEGs which are involved in the cell cycle on LN229 and Snb19. The DEGs were color coded, with the colors corresponding to the up- and down- expressed.

It has been demonstrated that DNA damage induced the cell cycle arrest in proliferating mammalian cells (Erasimus et al., 2016). At first, cell cycle progression was imaged using FUCCI fluorescent biosensor and microscopy. Different phases of the cell cycle were determined based on different fluorescence signals, red signal corresponding to G1 phase, yellow signal corresponding to G1/S phase and green signal corresponding to S/G2/M phase (Figure 5A). In this study, similar results were observed in both cell lines after the treatment. In DMSO condition, the highest number of cells were present in S/G2/M phase, moderate number of the cells were present in G1 phase, and least number of the cells were present in G1/S phase. Upon THTMP treatment, the majority of the cells were presence in G1 phase, following is the G1/S phase and small number of cells were in S/G2/M phase. In TMZ condition, the percentage of cells in different phases varied between G1/S and S/G2/M phase. According to these results, it is to conclude that GBM cells were arrested at G1/S phase when they were treated with THTMP and were arrested at S/G2/M phase when they were under TMZ treatment (Figure 5B).

Here, we show that THTMP induced the downregulation of many genes related to DNA replication, thereby, inhibiting the process of DNA replication and cell cycle progression. Next, genes associated with cell cycle progression were selectively analyzed (Figure 5C). There are several biological processes involved in cell cycle pathway that have been activated by the treatment. It includes cell cycle G1/S phase, G1/S transition of mitotic cell cycle, G2/M transition of mitotic cell cycle, cell cycle G2/M phase transition, cell cycle checkpoint and positive regulation of cell cycle (Figure 4A). Regarding the expression of various genes involved in cell cycle, genes in Snb19 have higher fold change compared to those in LN229 (Figure 5C). For example, the fold change of CCNA2 gene is -0.4 and -2.5 in LN229 and Snb19, respectively, when they were treated with THTMP. The fold change of CCNB2 gene is -0.3 and -2.1 in LN229 and Snb19, respectively, when they were treated with TMZ.

Here, the genes associated with G1 phase and G1/S checkpoint were first selectively analyzed (Figure 5C). CCNA2 gene coding to cyclin A2 protein was found to be downregulated in both cell lines when they were treated with THTMP. In Snb19, genes CCNE1 and CCNE2 coding to Cyclin E1 and E2 proteins were found to be decreased in THTMP treatment. It is noted that overexpression of Cyclin A and Cyclin E has the function to regulate G1/S transition when they complex with CDK2; thus, decreased expression level of CCNA2, CCNE1, and CCNE2 could lead to mediate the G1/S arrest. Moreover, BUB1 was identified to be downregulated in this study and was enriched in biological processes associated with the mitotic cell cycle, including cell cycle chromatid segregation, G1/S transition of mitotic cells and DNA replication. CDC20 appears to act as a regulator protein interacting with several other proteins at multiple points in the cell cycle. We found that CDC20 gene was downregulated and enriched in cell cycle and oocyte meiosis pathways.

In case of TMZ treatment, FUCCI analysis shows that both Snb19 and LN229 cells were arrested at S/G2/M phase. It is in accordance with our gene expression analysis. Genes CCNB1, CCNB2, CCNA2, which relate to Cyclin B1 and Cyclin A2, were found to be decreased. These two cyclins have the function to regulate G2/M transition when they complex with CDK1. Moreover, CDKN1A (p21) and GADD45A, two downstream target genes of p53 in the G2 checkpoint, were found to be increased in LN229 and Snb19, respectively, at the transcriptional level. Previous studies have reported that increased p21 expression led to the repression of cyclin B1 and Cdc2 promoters and that increased GADD45A expression inhibits Cdc2 activity, thereby mediating G2/M arrest (Jin et al., 2000; Yang et al., 2000).

The results show that THTMP induced cell cycle arrest at G1/S phase while TMZ induced cell cycle arrest at G2/M phase in both cell lines. This result implies that THTMP has inhibited synthesis of GBM cells before they can entry to replication and division periods; therefore, strongly preventing cell proliferation. Moreover, G1/S phase arrest of cell cycle progression provides an opportunity for cells to either undergo repair mechanisms or follow the apoptotic pathway (Bartek and Lukas, 2001).

### THTMP Increases ROS Production and Induces Pro-apoptotic and Anti-apoptotic Genes

**FIGURE 6:**
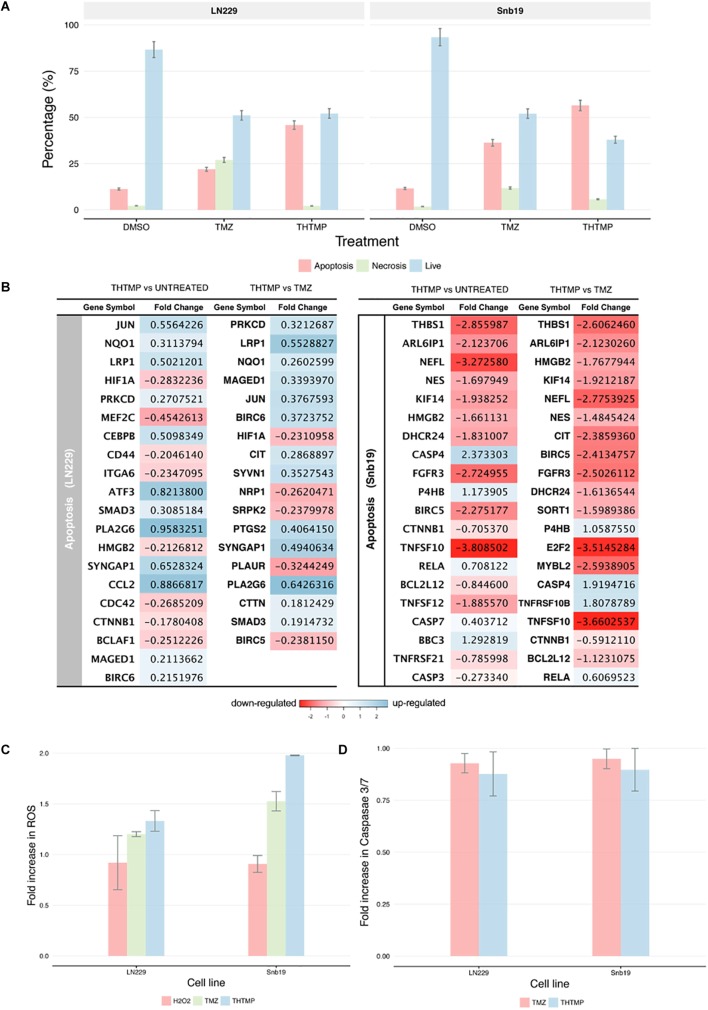
Apoptosis induction determination using double stains Annexin V and Propidium iodide and RNA-seq. **(A)** Percentage of apoptosis, necrosis and live cell using Dead Cell Apoptosis Kit with Annexin V-FITC and PI of untreated cell (DMSO control TMZ and compound THTMP at 24 h post-treatment on Snb19 and LN229. **(B)** The top 20 DEGs which are involved in apoptosis of LN229 and Snb19. The DEGs were color coded, with the colors corresponding to the up- and down- expressed. **(C)** Effect of THTMP and TMZ in intracellular ROS production. Fluorescence intensity of ROS was determined by activity of 2 μM H2DCFDA (30 min), fluorescent probe. H_2_O_2_ was used as the positive control. **(D)** Activity of caspases 3/7 in LN229 and Snb19. Caspase 3/7 was determined using luminescence plate reader. Fold increase in ROS and caspases 3 and 7 activity of LN229 and Snb19 cell lines was calculated when they were treated with THTMP and PC at IC_50_ concentration. Triplicates were performed for each condition.

Apoptosis induction assay was performed using Annexin V/PI double staining. Here, the percentage of apoptosis was calculated based on the cells with Annexin V-FITC positive and PI negative and both Annexin V-FITC and PI positive. The percentage of necrosis was defined based on the cells with Annexin V-FITC negative and PI positive (Chen et al., 2008). Figure 6A shows the live, apoptosis and necrosis of LN229 and Snb19 when they were treated with THTMP and TMZ. Generally, apoptosis induction was observed in both cell lines compared with positive control and untreated conditions. The apoptosis percentage of LN229 cells treated with THTMP is 45.8% while only 21.9 and 11.2% were obtained when they were treated with TMZ and DMSO, respectively. In case of Snb19 cells, 56.4% of apoptotic cells were found in THTMP treated condition whilst TMZ and untreated conditions exhibit only 36.2 and 11.5% apoptotic cells. Beside the apoptotic cells, necrotic cells were also observed in both cell lines. However, necrosis percentage is less than 10% in case of Snb19 while in LN229, 27.0 and 7.6% were found to be necrotic cells when they were treated with TMZ and THTMP, respectively.

The results above are in accordance with the gene expression profile showing the enrichment of apoptosis pathways including neuron apoptotic process, positive regulation of neuron apoptotic process, regulation of apoptotic signaling pathway, regulation of neuron apoptotic process, intrinsic apoptotic signaling pathway and extrinsic apoptotic signaling pathway. Genes involved in regulation of apoptotic process were presented in Figure 6B. Moreover, the gene expression profile indicates lower number of the DEGs related to apoptosis process in TMZ treatment compared to THTMP treatment (Figure 6B). The expression changes showing in Figure 6B revealed that THTMP tended to induce pro-apoptotic genes, reduce anti-apoptotic genes and also induce some anti-apoptotic genes.

Among pro-apoptotic genes, CTNNB1 gene coding for β-catenin protein was downregulated in LN229 cells when they were treated with THTMP. It is reported that abnormal accumulation of β-catenin contributes to most cancers and repressed CTNNB1 also leads to inducing apoptosis in some tumor cells (Yang et al., 2017). Interestingly, the pro-apoptotic Bcl-2 family gene MAGED1 was also found to be upregulated in LN229 whereas BBC3 (PUMA) gene was upregulated in Snb19 when they were treated with THTMP. Moreover, BCL2L12, an anti-apoptotic gene, was found to be downregulated in Snb19 cells. It is noted that BCL2L12 expression is upregulated in most human glioblastomas. Expression of Bcl2L12 results in resistance to apoptosis (Yang et al., 2015). Our findings demonstrated that THTMP has shown the ability to induce apoptosis of Snb19 and LN229 via mitochondrial pathway throughout the upregulation of pro-apoptotic and downregulation of anti-apoptotic Bcl-2 family genes.

Although the altered expression of genes described above could confirm apoptosis, genes involved in anti-apoptosis were expressed when the cells were treated with THTMP. The anti-apoptotic characteristics of Snb19 cells were identified by the downregulation of several genes from the membrane stress receptors, such as TNFSF10, TNFSF12, and TNFRSF2. Moreover, the upregulation of RELA, a member of NFkB family, suggested a decline in inflammatory processes and strong anti-apoptotic properties for this cell line. In LN229 cells, the regulation of the TNF receptor pathway as well as NFkB signaling pathway was not significantly affected, but there was a modest upregulation of BIRC6 encoded for BIRC protein, a member of the inhibitor of apoptosis (IAP) gene family preventing apoptotic cell death. Interestingly, the BIRC5 was suppressed in Snb19.

In addition to the activation of apoptotic pathways in the treated cells, reactive oxygen species (ROS) could lead to cell cycle arrest and induces apoptosis in anticancer treatment (Circu and Aw, 2010). It is well known that ROS is produced in both normal and abnormal cells especially in cancer cells. ROS plays an important role in proliferation, survival, metastasis and angiogenesis (Clerkin et al., 2008). In this study, the effects of THTMP, TMZ and H_2_O_2_, a positive control in the levels of ROS on GBM cells, was assessed using ROS production assay. Figure 6C shows an increase of ROS level when the cells were treated with THTMP and TMZ. Interestingly, the fold increase of ROS of Snb19 and LN229 cells treated with THTMP were higher than H_2_O_2_. As seen in Figure 6C, Snb19 cells have higher level of ROS compared to LN229 in all of the conditions. In which, up to 2 fold increase was found in THTMP treatment of Snb19 while only 1.3 fold increase was observed in LN229. The same trend was observed for the TMZ treatment in which 1.5 fold and 1.2 fold were obtained in Snb19 and LN229, respectively. Thus, ROS were significantly produced when GBM cells were affected with THTMP. This suggests that higher ROS level could be interlinked with the observed apoptotic cell death of cancer cells upon treatment with compound THTMP. Moreover, in mammalian cells, ROS are produced by normal oxidative metabolism and cellular antioxidants such as superoxide dismutase (SOD1) and thioredoxin (TRX1) detoxify these species (Covarrubias et al., 2008). A study indicated that decreasing SOD1 and TRX1 could lead to apoptosis induction in glioma cells when they were treated with kaempferol, a natural phenolic compound, via elevation of ROS (Sharma et al., 2007). In agreement, we also found out that while THTMP treatment had no effect on TRX1, a decrease in SOD1 was observed in Snb19 cells (Supplementary Table S3). Thus, THTMP induced ROS-mediated apoptosis.

According to Figure 6D, caspase 3/7 was not significantly increased in both cell lines when they were treated with THTMP and TMZ. This is also in agreement with the gene expression profile where we could not find the significant expression of CASP3 and CASP7 in LN229 cells (Figure 6B). In case of Snb19 cells, although the CASP3 and CASP7 were expressed, the CASP3 was downregulated and CASP7 was upregulated when they were treated with THTMP (Figure 6B). This result interprets the no change in caspase 3/7 activation assay results (Figure 6C). The repression of caspase genes might be caused from the activation of anti-apoptotic genes (Stegh et al., 2008). Thus, apoptosis inductions of GBM cells by THTMP and TMZ were not via caspase 3/7. Instead of CASP3 and CASP7, CASP4 was found to be upregulated in Snb19 when they were treated with both THTMP and TMZ. Therefore, the result implies the involvement of caspase 4 in Snb19 when they were treated with THTMP and TMZ.

## Discussion

DNA damage response induced by THTMP was validated and shown to be dose-and time-dependent. A detailed analysis of global molecular expression profiling is required in order to understand the complex cellular responses. Here, by combining biochemical studies with high-throughput RNA sequencing, the changes in gene expression in glioma cells induced by THTMP was explored. Our results show that genes involves in DNA damage, DNA replication, cell cycle arrest and apoptosis induction were transcriptionally modulated and highly enriched when GBM cells were treated with THTMP.

Firstly, our study demonstrated a THTMP induced activation of various genes associated with DNA damage and cell cycle arrest. The cell cycle arrest in GBM when they were treated with THTMP and TMZ may be explained by transcriptional expression change of some crucial genes that are listed in Figure 5B. CCNA2 gene was found in both Snb19 and LN229 when they were treated with THTMP compound. This gene belongs to a highly conserved cyclin family and the encoded protein of this gene is crucial in the control of the cell cycle at G1/S and G2/M transition points. Here, several important genes in control cell cycle at G1/2 and G2/M such as CCNA2, BUB1, CDC20 are selected for further discussion in order to understand their mechanism in controlling cell cycle of GBM.

In previous studies, it is reported that overexpression of CCNA2 is involved in tumor transformation and progression in numerous types of cancer (Uhlen et al., 2010). As expected, our results show that CCNA2 was downregulated, which is in accordance with the function of cyclin A2 protein in cell cycle indicating that CCNA2 inhibits the growth of GBM. BUB1 was also found to be downregulated that can explain the G1/S transition arrest since the BUB family of genes encode proteins that are involved in large multi-protein kinetochore complex, and are reported to be key component of the checkpoint regulator pathway. BUB1 encodes a serine/threonine protein kinase which plays an important role in mitosis (Tang et al., 2006), and BUB1 accumulates at unattached kinetochores where it mediates the recruitment of mitotic arrest deficient (Mad) dimers. Combination of Mad and BUB1 leads to prevention of premature separation of sister chromatids until all chromosomes are correctly attached to kinetochores; thus, correctly chromosome segregation achieved (Ricke et al., 2011). This suggests that GBM cell growth may be inhibited by regulating the mitotic cell cycle in THTMP treated conditions (Grabsch et al., 2003). In addition, previous reports indicate that CDC20 is highly expressed in various type of human tumors including breast, cervical and glioblastoma cancer (Marucci et al., 2008; Jiang et al., 2011; Rajkumar et al., 2011). It is also reported that expression level of CDC20 is correlated with the grade of glioblastoma and it is expressed at different levels in patients at different ages (Bie et al., 2011). In the study CDC20 was downregulated, which leads to conclude that CDC20 may inhibit GBM growth. According to the biological process enrichment results, CCNA2 was enriched in cell cycle and oocyte meiosis pathways, in which CDK1, BUB1 and CDC20 were also involved. These genes are also known as key genes playing a crucial role in promoting GBM growth (Chen et al., 2016). Repression of these genes when the cells were treated with THTMP indicates that this compound strongly inhibits the growth of GBM throughout cell cycle arrest.

Secondly, we detected apoptotic effects induced by THTMP. Analysis of apoptosis genes subsequently revealed that the progress of apoptosis was accompanied by changes in both pro-apoptotic and anti-apoptotic gene expression, consistent with the observation in other chemotherapeutic therapies in cancer cells (Kim et al., 2003). It is noted that BCL2L12 is overexpressed in primary GBM and functions to inhibit post-mitochondrial apoptosis signaling (Stegh et al., 2007). This study shows that THTMP has induced apoptosis via mitochondrial pathway in both LN229 and Snb19 cell lines due to the suppression of Bcl-2 family, BCL2L12. The study also shows that a large number of genes of Snb19 was expressed compared to LN229 when they were treated with THTMP. The anti-apoptotic genes expressed in LN229 and Snb19 are different. This result implies that apoptosis pathways of GBM cells will be executed in different mechanisms when they were treated with THTMP. This study shows that TMZ also induced apoptosis in GBM cells as explored by Annexin V/PI double staining; however, the gene profile of this condition is still limited. Here, the absence of caspase 3/7 activation indicates that THTMP has not induced apoptosis via caspase 3 and 7 of LN229 and Snb19, but the caspase 4 might be involved in the apoptosis pathway of Snb19 cells.

## Conclusion

Overall, the effect of THTMP on GBM cells is relatively much stronger than TMZ in all aspects. This study provides experimental evidence that THTMP is capable of inhibiting the growth of GBM cells. THTMP has the ability to induce DNA damage through the p53 signaling pathway leading to cell cycle arrest. The G1/S checkpoint arrest depends on the decrease of cyclin A2 in both Snb19 and LN229 cells-treated with THTMP. Moreover, the decrease of cyclin E1 and E2 in Snb19 in THTMP treatment also contributes to the G1/S checkpoint arrest. This result suggests that THTMP facilitates cancer cells to undergo programmed cell death pathways, apoptosis in glioblastoma cells. The induction of apoptosis of GBM in THTMP treated conditions is associated with increasing pro-apoptotic factor of Bcl-2. In addition, it is indicated that caspase 4 may play a role in this apoptosis induction instead of caspase 3/7.

The findings on inhibition of GBM proliferation and downregulation of cell cycle genes in the G1/S phase not only provide a better understanding of the mechanisms of THTMP, a phenolic compound, as anticancer agent, but also open an avenue for investigating the role of oxidative stress in GBM involving cell cycle and apoptosis regulation. However, testing THTMP on glioma animal model and computational pharmacogenomics approaches (Musa et al., 2018) will allow this compound to be used as a potential chemotherapeutic drug for glioma treatment.

## Author Contributions

PD executed experiments and data analysis. AM analyzed the RNAseq data. NC prepared and characterized the compounds. FE-S contributed in development of the project. OY-H contributed in development of the project, conceived and managed the project. MK designed and supervised the experiments of the biological assays, data analysis, conceived and managed the project. All authors involved in the manuscript write up and approved the final version of the manuscript.

## Conflict of Interest Statement

The authors declare that the research was conducted in the absence of any commercial or financial relationships that could be construed as a potential conflict of interest.

## Footnotes

1 ftp://ftp.ensembl.org/pub/release-92/fasta/homo_sapiens/dna/

2 ftp://ftp.ensembl.org/pub/release-92/gtf/homo_sapiens

3 https://wiki.eduuni.fi/display/tutsgn/TUT+Narvi+Cluster

4 https://bioconductor.org/packages/release/bioc/vignettes/DESeq2/inst/doc/DESeq2.html

5 http://www.pantherdb.org/, released on October 24, 2016
